# Dr. Scouler on the Production of Worms in the Human Body

**Published:** 1830-07-01

**Authors:** 


XXVI.

### On the Production of Worms in the Human Body. By J. Scouler, M.D. &c. Professor of Natural History in the Andersonian University.

A very ingenious paper on the above intricate subject is published in the May number of the Glasgow Medical Journal, from which we are tempted to make an extract. The formation of worms in the human body is one of the most difficult problems in physiology—and is one of considerable interest even to the practical physician. We do not deem it necessary to go over the various hypotheses that have been framed, from that of "equivocal generation," maintained by the ancients, down to the revival of the same hypothesis by Blumenbach, Rudolphi, and other German naturalists. It is a startling doctrine! We have the authority of Holy Writ that man himself is only a worm;—and if worms are produced by equivocal generation—or, in other words, by the "fortuitous concourse of atoms," it is needless to suppose that the act of the Deity was necessary for man's first creation from the earth on which he crawls! With the hope of releasing us from this humiliating supposition, we gladly introduce to our readers the lucubrations of Dr. Scouler. After a critical analysis of the doctrines maintained by ancients and moderns on this subject, he thus develops his own ideas.

"We have now given a very brief outline of the theories by which the origin of worms has been attempted to be explained; but enough has been stated to prove that every one of them is utterly at variance with well-known facts. One theory yet remains, founded on very different principles, and which demands our most candid consideration.

Do worms originate from a spontaneous generation? Generation, or the production of a living being, consists, if we separate it from all accidental circumstances, in the separation of a portion of the parent animal, which is endowed with independent vitality. This portion is formed in the ovarium of the female before she has any intercourse with the male, and the principal effect of the spermatic fluid is to excite the dormant powers of the germ into action, and to modify its form. This is the essence of generation properly so called; but there is another way in which animals, or parts of animals, may be produced. If we cut off the leg, or extirpate the eye, of a water newt, these parts are again restored after the interval of a few months, and only differ from those which had been destroyed in being a little smaller. The claws of lobsters and the rays of star fish are reproduced in the same manner. In the lower animals, this process reaches its maximum of development, and becomes a new mode of continuing the species. If we cut a polype into two portions, and the circumstances be favourable, each section becomes a perfect animal. Many worms, if divided in this manner, grow under the knife, and, if abundance of food be supplied, the division may be continued ad infinitum. A very common fresh water worm, the Planarea of naturalists, if well supplied with food, exhibits this process spontaneously. The animal begins to swell, and several fissures appear on its surface, and it gradually separates into several distinct portions, each of them becoming a perfect animal. Hence generation, by means of sexes, is not necessary for the continuation of animals, and the law of Harvey, *omne vivum ex ovo*, must be changed into *omne vivum e vivo*. This mode of reproduction is by no means rare among the simpler animals; indeed it appears to be the only one by which the infusory animalcules are propagated.

Having proved that generation, by means of two sexes, is not essential for the propagation of many animals, we are prepared to advance a step further, and maintain that entozoa may be produced by the animal which they inhabit. When we consider the extreme complexity of the organization of the more perfect animals, and vast variety of tissues which enter into the composition of their organs, we may grant that it is not impossible that the vessels which erect such delicately constituted parts may form others of far greater simplicity. Thus, in many diseases, tumours and tubercles are formed in the organs of animals ; and these new formations possess a structure as different from any of the regular tissues of the animal which produced them, as the texture of an entozoon does from that of the creature it inhabits. These tumours may be considered as possessing a degree of vitality peculiar to themselves, and only deriving their nourishment from the body to which they are attached. In the same manner, after rupture of the uterus, when, fortunately for the parent, she survives the accident, the fœtus becomes encysted, we have a phenomenon of the same kind, the fœtus retaining a portion of its vitality, and the requisite nourishment is derived from the vessels of the mother.

In the instance of tumours and tubercles, already alluded to, their origin is doubtless to be accounted for from a deranged action of the assimilatory arteries, which is of two kinds ; in the first they produce a formation which is analogous to the regular structure of some other part of the body, as ossifications and steatomatous tumours. When nutrition is still more deranged, parts are formed which possess no analogue whatever in the healthy state.

Now, it is not improbable that many entozoa owe their origin to a process similar to that which we have described. Worms are always a consequence of a diseased state, and when they produce bad symptoms, it is from some accidental circumstance depending on their size, numbers, movements, or from the importance of the organ in which they are found. So that as there is a diathesis peculiar to most organic diseases, there is also a verminous diathesis. In support of this, it may also be added, that worms differ from all other animals in being intimately dependant on the organization which they inhabit for the continuation of their existence ; and, in this respect, the parallel between encysted worms and the encysted fœtus, already mentioned, is perfect in every point of view. As the mode of nutrition in every animal is, to a certain degree, *sui generis,* so the worms which they produce are also peculiar.

To express clearly the manner in which worms are supposed to be formed, and to prevent mistakes, we will condense what has been said in a few words. It is not maintained that entozoa are produced from the fluids found in the intestinal tube or in the tissue of organs, but that their germs are formed by some aberration of nutrition, in a manner similar to that in which other new formations originate, and that this germ becomes endowed with an independent vitality, and is evolved into a worm.

The consideration of hydatids affords us a proof of the manner in which entozoa are formed. These cysts are of two kinds, those which are mere serous vesicles, and those which are true entozoa; but the transition from the one to the other is very gradual. We first observe a serous cyst smooth, polished within, and containing a limpid fluid, and exteriorly fibrous. The next kind, the Acephalocysts of Laennec, consist of an internal fibrous capsule, containing a cyst, which is very thin and transparent, filled with a fluid, but destitute of a head or any distinct organs ; these are intermediate between the cysts of the first kind and the true entozoa ; so that some writers include them among the common hydatids and others among the entozoa. We have next the Echinococcus, which is included in a fibrous cyst like the preceding ones, but contains in its interior many little bodies, which are acknowledged by all writers to be true worms. From these the transition is easy to be cysticerci, with a head like that of a tape worm, attached to a transparent cyst, included, as usual, in a fibrous one. Lastly, we have often found round worms included in such cysts. These facts afford a strong support to the theory we have adopted.

There are several objections which may be urged against this theory which it is necessary to notice. As many entozoa have distinct organs of generation, and some of them have individuals of both sexes, and possess a very complicated organization, it has, with great propriety been urged, that there is no need for seeking any other way of accounting for their propagation than the usual one, and that it is contrary to every analogy to attribute their formation to the process we have described.

To this remark it may be replied, that it has already been demonstrated that the ova of entozoa cannot travel through the tortuous paths of the circulatory vessels; and, further, many worms, as the hydatids for example, possess no genital apparatus. Nor is it to be ascertained that, if the theory we have advanced be correct, organs of generation are of no use; for, if a worm be once formed, it then propagates its species in the usual manner. A still greater alleviation of this objection is derived from the fact, that many animals, endowed with organs of generation, are frequently propagated by a process totally distinct from it, namely, by a division of their bodies.

Another objection is that, if entozoa were produced in the manner described, we should have nothing regular and determined in their forms, but that new animals of monstrous forms and confused organization would be daily arising.

Nature, however, leaves nothing vague and undetermined in her works; and the production of entozoa is regulated by fixed and unchanging laws. The same modification in the nutrition of an organ, which produced the first intestinal worm, still continues to operate, and consequently the same animal is formed. We might as well expect to have new diseases springing up every day, and that laws, which regulate the formation of a cancer or an exostosis, should be different in every individual. Nature is always uniform in her operations, and even her most irregular productions are governed by regular laws. Even monsters, which, for a long time, were supposed to be the production of some fortuitous concourse of circumstances, can now be classified and described, and the laws of their formation ascertained with as much precision as those which preside at the origin of a regularly organized individual.

A third objection which may be urged is, that according to this theory, if worms are produced in the manner it supposes, we should have some regular apparatus of a temporary nature, at least, set apart for their production.

We may reply to this argument, that there are many parts formed in the bodies of animals differing completely from those which are found while it is in a healthy state, and yet we see no apparatus by which they are called into being. We see no apparatus for the production of encysted tumours, or for the formation of earthy matter in the valves of the heart. In these instances, as in the origin of entozoa, we have merely to attribute all these phenomena to the aberrations of nutrition, which constitutes the essence of every disease, and the chief cause of every organic change."

We beg to return our thanks to Dr. Scouler for the pleasure which we have derived from a perusal of his interesting paper.

---

